# Rare Presentation and Diagnostic Challenge of a Germ Cell Tumor of the Yolk Sac Type

**DOI:** 10.7759/cureus.53138

**Published:** 2024-01-29

**Authors:** Inês Gomes, Emanuel Jesus, Gabriela M Sousa

**Affiliations:** 1 Medical Oncology, Instituto Português de Oncologia de Coimbra Francisco Gentil, Coimbra, PRT

**Keywords:** yolk sac tumor, chemotherapy, neurologic disorder, opsoclonus-ataxia, germ cell tumor

## Abstract

Opsoclonus-ataxia paraneoplastic syndrome (OAPS) is a rare neurological disorder often associated with malignancies. This case report highlights an unusual instance of OAPS linked to a yolk sac (germ cell) tumor, a correlation underrepresented in the medical literature. The patient presented with distinct neurological symptoms alongside mediastinal lymphadenopathies. The subsequent diagnostic journey revealed a yolk sac germ cell tumor. Following incisional biopsies and treatment, the patient experienced fluctuations in mental status, leading to challenges in initiating chemotherapy. Despite these complications, a multidisciplinary approach involving neurologists, oncologists, and hematologists was pivotal. The case emphasizes the complexities of managing OAPS in tandem with a germ cell tumor, underscoring the need for further research and highlighting the significance of specialized neurological evaluation in similar cases.

## Introduction

Opsoclonus-ataxia paraneoplastic syndrome (OAPS) is an infrequent (0.1-10% of cancer patients) neurological disorder characterized by the concurrent presence of distinct symptoms: rapid, uncontrolled eye movements called opsoclonus, and disrupted coordination of voluntary movements referred to as ataxia [1,2]. OAPS emerges through two predominant etiological pathways, with a primary emphasis on the paraneoplastic and idiopathic origins [3]. The paraneoplastic pathway underscores the syndrome's intricate association with an immune-mediated cascade, set in motion by the host's immune system targeting its own neural constituents. This immunological reaction is elicited in direct response to the presence of an underlying neoplastic ailment [4].

Cases of OAPS have been documented in association with malignant tumors, including neuroblastoma (predominantly in pediatric patients), small-cell lung cancer, breast cancer, and, in rare instances, gynecological cancers [5]. The correlation of this syndrome with germ cell tumors is notably uncommon and inadequately elucidated in the available literature. This article presents a clinical case involving a germ cell tumor, wherein the primary clinical presentation manifested as OAPS.

## Case presentation

**Figure 1:**
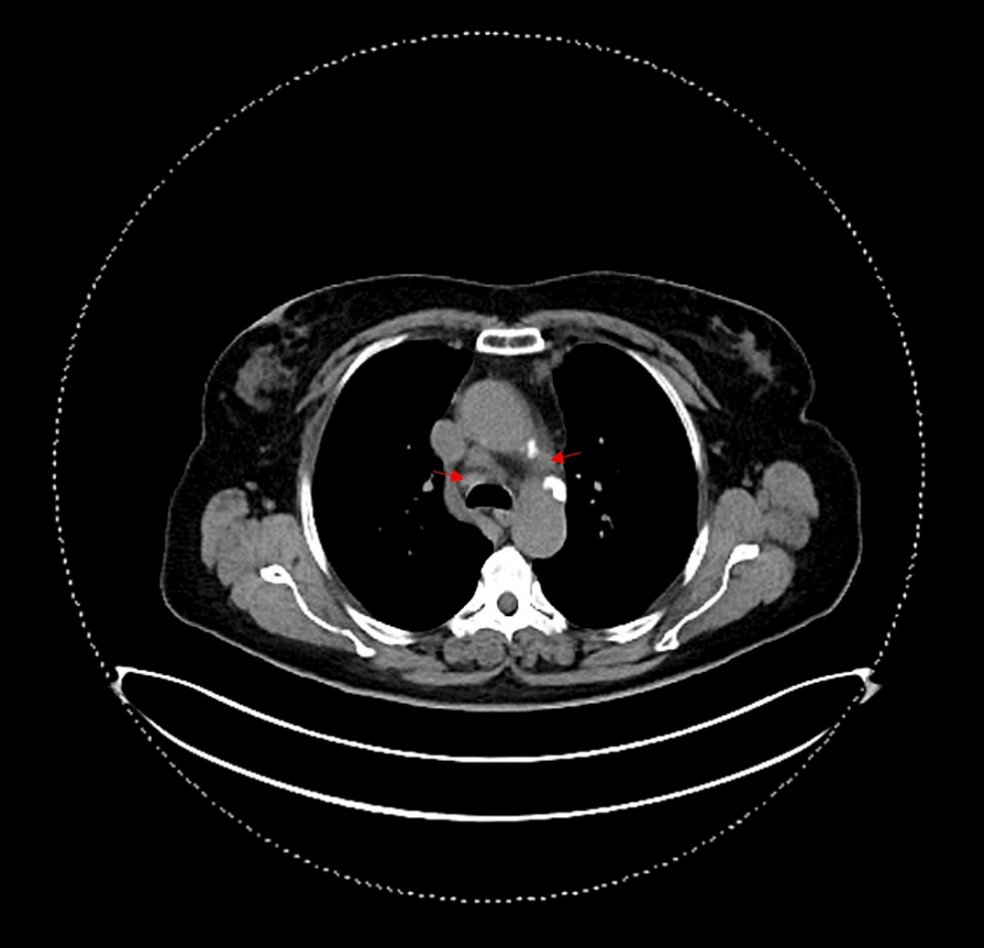
Thoracic CT Red arrows - mediastinal lymphadenopathies

A 63-year-old female patient, with a history of smoking (47 pack-years over 34 years), chronic obstructive pulmonary disease, hypothyroidism due to total thyroidectomy 20 years ago (benign thyroid nodules), and ocular surgery due to a childhood accident. In April 2022, she developed haemoptoic sputum. In this context, a chest computed tomography scan (CT) was performed, revealing mediastinal lymphadenopathies, and left internal mammary chain lymphadenopathies (Figure 1). The positron emission tomography-computed tomography scan (PET-CT) from 12 July 2022 showed multiple hypermetabolic right bronchial-hilar lymphadenopathies, most prominently in relation to the superior lobar bronchus, with ill-defined borders, measuring about 13x9mm in its largest axes (SUVmax 14.3); and mediastinal lymphadenopathies (upper para-sternal, upper and lower para-tracheal on the right, pre- and sub-carinal), most prominently in the left upper para-sternal region, measuring 25x16mm (SUVmax 13.2) and sub-carinal with 19x15mm (SUVmax 10), consistent with pulmonary metastasis. An endobronchial ultrasound-guided aspirate biopsy on 18 July 2022 showed morphological and immunophenotypic characteristics of non-Hodgkin B-cell lymphoma, large cells with a germinal centre immunophenotype (predominantly strongly reactive cells for CD10, with immunoreactivity for B-cell lymphoma 2 (BCL2) in about 20%, for B-cell lymphoma 6 (BCL6) in more than 30%, and also for cellular Myc (C-MYC) in about 20%; no immunoreactivity for multiple myeloma oncogene-1 (MUM1). In view of the result but given that the type of biopsy performed (aspiration puncture) could not be predictive of the final diagnosis, an incisional biopsy of the lesion in the left internal mammary chain was conducted on 14 September 2022, whose result coincided with a germ cell tumour of the yolk sac type. Histology results showed medium or large cells, with nuclei showing obvious pleomorphism and sometimes with small nucleoli, with apparent pseudoinclusions ("towel" distribution). In the immunohistochemical study, the neoplastic cells showed strong and diffuse positivity for glypican, heterogeneous positivity for caudal-type homeobox 2 (CDX2) and cluster of differentiation 99 (CD99) and focal positivity for anti-cytokeratin monoclonal antibodies 1 and 3 (AE1/AE3), cell marque cytokeratin (CAM 5.2) and epithelial membrane antigen (EMA); negativity was observed for alpha-fetoprotein, tyrosine-protein kinase KIT (C-Kit), human leukocyte common antigen (LCA), cytokeratin 7 and 20 (CK7/CK20), thyroid transcription factor 1 (TTF1), inhibin, cluster of differentiate 20 (CD20), cluster of differentiate 3 (CD3) and cluster of differentiate 30 (CD30); Ki-67 of 80%.) 

Three days after discharge, she developed vertigo, tremors, and vomiting, and went to the local hospital's emergency room. Neurological examination revealed opsoclonus overlaid with alternating flutter in the superior nystagmus, with right, left, upper, and lower eccentricity.

From the conducted study, analytically, no significant alterations were observed in the complete blood count, biochemistry (including renal function, electrolytes, liver function), coagulation, and thyroid function tests (Table 1).

**Table 1:** Blood test results

Blood Test	Result	Units	Reference values
Leucocytes	6.7	10^3^/μL	4.0 - 11.0
Neutrophiles	4.5	10^3^/μL	1.8 - 7.0
Lymphocytes	1.1	10^3^/μL	1.0 - 5.0
Hemoglobin	13.2	g/dL	12.0 - 16.0
Platelets	288	10^3^/μL	140 - 400
Fasting glucose	121	mg/dL	70 - 115
Urea	32	mg/dL	10 - 50
Creatinine	0.9	mg/dl	0.5 - 1.2
Sodium	135	mg/dl	135 - 145
Potassium	3.6	mmol/L	3.5 - 5.1
Ionized calcium	1.13	mmol/L	1.14 - 1.29
Aminotransferase, alanine	32	U/L	≤40
Aminotransferase, aspartate	30	U/L	≤40
Gamma-glutamyl transpeptidase	50	U/L	≤49
Total bilirubin	0.3	mg/dL	≤1.2
Direct bilirubin	0.1	mg/dL	≤0.3
Lactate dehydrogenase	630	U/L	120 - 246
Albumin	3.5	g/dL	3.5 - 5.0
Protein, total	5.9	g/dL	6.0 - 8.0
Parathyroid hormone	73.0	pg/mL	18.0-80.0
25-Hydroxycholecalciferol	28.56	ng/mL	18 - 80
Thyroid-stimulating hormone	3.2	μUI/mL	<4.5
Antinuclear antibodies	0	units	≤1.0
Anti–smooth muscle antibodies	0	units	≤ 1:80
Antineutrophil cytoplasmic antibodies	0	units	Negative

An electroencephalogram on 21 September 2022 showed preserved background activity with no pathological activity, particularly no slow activity in the temporal regions. Immediate performance of cranioencephalic magnetic resonance imaging (CE-MRI) was not possible due to patient non-cooperation, but the cranial CT showed left anterior temporal venous flow void and severe leukoaraiosis.

During hospitalization in neurology, she received five days of human immunoglobulin and then oral prednisolone 50 mg per day, leading to clinical stabilization. At the time of transfer to the oncology service, she still had ocular opsoclonus and superior nystagmus.

A lumbar puncture was performed (clear and slightly cloudy cerebrospinal fluid), ruling out viral aetiology, no alteration in cell count, protein or glucose and not showing neoplastic cells (Table 2). While in the oncology unit, she continued treatment with prednisolone, with a gradual tapering. The search for autoantibodies in the blood as part of the immune study showed negative results.

**Table 2:** cerebrospinal fluid results WBC, white blood cells

Cerebrospinal fluid	Result	Units	Reference values
Cell count	1	WBC/mm^3^	≤5
Glucose	53	mg/dL	40 - 80
Total proteins	45	mg/dl	15 - 60

The patient showed progressive improvement of the neurological condition with corticosteroid therapy, although initiation of chemotherapy was delayed due to fluctuations in mental status, evaluated with the confusion assessment method (CAM), including periods of agitation interspersed with worsening drowsiness. After therapeutic adjustments, including prednisolone dose (20 + 20 mg), she started the first cycle of chemotherapy with the BEP regimen (bleomycin 30000 IU Days 2, 8 and 15 + cisplatin 50 mg/m2 on Days 1 and 2 + etoposide 165 mg/m2 on Days 1, 2 and 3) on 7 November 2022. On Day 8 of the cycle, she developed grade 2 mucositis and tested positive for SARS-CoV-2, without specific symptoms. On Day 10 of the cycle, she developed a fever associated with grade 4 neutropenia (absolute neutrophil count 300/μL), grade 2 diarrhoea and septic shock, requiring noradrenaline support. A microbiological study revealed the presence of Escherichia coli in blood cultures, and the patient received antibiotic therapy with piperacillin/tazobactam, adjusted according to antibiotic sensitivity testing. Additionally, she received a platelet transfusion due to grade 4 thrombocytopenia (12000 103/μL), based on CTCAE v5.0. Gradual tapering of aminergic support was performed with good clinical and analytical evolution.

One month after the BEP cycle, considering clinical improvement in terms of opsoclonus (currently resolved), as well as clinical stability and resolution of febrile neutropenia, the patient was discharged to a long-term care facility. Due to the severity of the infectious condition after neutropenia secondary to chemotherapy, the multidisciplinary team decided not to administer further chemotherapy. The patient remained under surveillance, exclusively managed by the palliative care team, and eventually died on 24 January 2023.

## Discussion

The rarity of the association between OAPS and germ cell tumours is a notable factor contributing to the limited clinical guidance on its management [6]. This case emphasizes the diverse neurological presentations that can accompany neoplastic processes, adding complexity to our understanding of OAPS. While OAPS is more commonly associated with malignancies like neuroblastoma, small-cell lung cancer, and breast cancer, its occurrence alongside germ cell tumours remains inadequately understood [6].

The intricate interplay between the immune system and neural constituents in paraneoplastic syndromes such as OAPS has become a pivotal focus of research [3]. In this specific case, the immune system's abnormal response to the underlying germ cell tumour likely played a role in the development of opsoclonus and ataxia. Unravelling these immunological mechanisms is crucial for comprehending the pathophysiology of paraneoplastic syndromes and developing targeted immunomodulatory therapies [2,3]. In the present case, we couldn't identify any alterations in the autoimmunity panel conducted; however, the patient's opsoclonus symptoms resolved with corticoid therapy after one cycle of chemotherapy. In other case reports documented in the literature, clinical improvement was observed upon initiating corticoid therapy, and the analytical assessment did not consistently reveal changes in antibody values [1, 5, 7].

The decision to administer chemotherapy was met with unforeseen complications, necessitating a balance between aggressive oncological intervention and the patient's overall well-being. The subsequent infectious complications, including SARS-CoV-2 infection, neutropenia, and septic shock, further exemplify the challenges faced in such cases. A multidisciplinary approach played a crucial role in guiding this patient's care. Collaboration between neurologists, oncologists, haematologists, and infectious disease specialists was instrumental in tailoring treatment strategies.

Additionally, this case highlights the importance of vigilance for atypical neurological presentations in patients with germ cell tumours, prompting consideration for early intervention and specialized neurological evaluation.

## Conclusions

This case offers a compelling illustration of the complexities inherent in the management of OAPS associated with a germ cell tumour. It underscores the critical role of a multidisciplinary approach and highlights the need for continued research into the underlying immunological and neurological mechanisms driving such paraneoplastic syndromes. By expanding our understanding of these rare clinical intersections, we can ultimately improve outcomes and quality of life for patients facing similar diagnostic and therapeutic challenges.

## References

[1] Nakajima M, Uchibori A, Ogawa Y (2018). CV2/CRMP5-antibody-related paraneoplastic optic neuropathy associated with small-cell lung cancer. Intern Med.

[2] Bataller L, Graus F, Saiz A, Vilchez JJ (2001). Clinical outcome in adult onset idiopathic or paraneoplastic opsoclonus-myoclonus. Brain.

[3] Armangué T, Sabater L, Torres-Vega E (2016). Clinical and immunological features of opsoclonus-myoclonus syndrome in the era of neuronal cell surface antibodies. JAMA Neurol.

[4] Bataller L, Rosenfeld MR, Graus F, Vilchez JJ, Cheung NK, Dalmau J (2003). Autoantigen diversity in the opsoclonus-myoclonus syndrome. Ann Neurol.

[5] Kanno K, Kin S, Hirose M, Suzuki S, Watanabe T, Fujimori K (2015). Opsoclonus-ataxia syndrome associated with ovarian mature teratoma. J Obstet Gynaecol Res.

[6] Klaas JP, Ahlskog JE, Pittock SJ (2012). Adult-onset opsoclonus-myoclonus syndrome. Arch Neurol.

[7] Webster EM, Tymon-Rosario J, D'Addario J, Zeybek B, Ratner ES (2020). Opsoclonus-ataxia syndrome and mature ovarian teratoma in an adolescent. Gynecol Oncol Rep.

